# Social determinants are associated with clinical presentation of acute pathological fracture in metastatic long-bone disease

**DOI:** 10.1016/j.jbo.2025.100707

**Published:** 2025-08-05

**Authors:** Tom M. de Groot, Lotte R. van der Linden, Angad D.S. Bedi, Andreea A. Lucaciu, Caleb C. Jang, Olivier Q. Groot, Job N. Doornberg, Paul C. Jutte, Santiago A. Lozano-Calderon, J.H. Schwab

**Affiliations:** a Department of Orthopedic Surgery, Massachusetts General Hospital, Boston, MA, USA; b Department of Orthopedic Surgery, University Medical Center Groningen, Groningen, The Netherlands; c Department of Orthopedic Surgery, University Medical Center Utrecht, Utrecht, The Netherlands; d Department of Orthopedic Surgery, Cedars Sinai Health Center, Los Angeles, CA, USA

**Keywords:** Metastatic long-bone disease, Social determinants, Pathologic fractures, Survival, Long-bone

## Abstract

**Background:**

Social Determinants of Health (SDOH) are non-medical factors that influence health, which have gained recognition across medical disciplines. Their impact on survival and disease presentation of patients with metastatic bone disease (MBD) remains unexplored.

**Methods:**

This retrospective observational study included 712 undergoing surgery for symptomatic long-bone metastases patients between 2013 and 2022. SDOH were evaluated using Cox Proportional hazards regression for post-operative survival. A multivariate logistic regression analysis was performed to identify associated factors for clinical presentation with a completed pathologic fracture.

**Results:**

The median overall survival was 264 days (IQR 74–772). Clinical presentation with a pathologic fracture as the initial symptom of metastatic bone disease (MBD) was observed in 15 % of patients (106/712).

SDOH factors played a significant role in clinical presentation. Patients with secondary insurance coverage were substantially less likely to present with a pathologic fracture (OR 0.26, 95 % CI 0.14–0.49; p < 0.01). In a sub-analysis of the most common tumors (breast, renal, and lung cancer patients; n = 353), attending college was associated with a significantly lower likelihood of presenting with a pathologic fracture as the initial symptom of metastatic bone disease (OR 0.54, 95 % CI 0.30–0.95; p = 0.03)

**Conclusion:**

This study suggests that unfavorable SDOH factors are associated with decreased post-operative survival and a higher likelihood of initial clinical presentation with a completed pathological fracture. Incorporating social determinants into comprehensive care strategies for individuals with MBD may guide targeted interventions and optimize patient management to improve outcomes.

## Highlights

• 15 % of patients present with a pathological fracture as first sign of bone metastases.
• Being single or unemployed is linked to worse survival after surgery for bone metastases.
• Lower education and limited insurance is linked to pathologic fracture as first symptom of bone metastases.
• Social factors can help predict survival and fracture risk in metastatic bone cancer.

## 1 Introduction

The risk of pathologic fractures increases when cancer metastasizes to weightbearing bones. Prophylactic stabilization of these impending fractures appears to have better outcomes than surgically treating pathological fractures in an acute setting [1]. Unfortunately, current efforts to predict pathological fractures in metastatic bone disease (MBD) have been limited by the lack of accuracy [2] or non-user-friendly CT-based models [[3], [4], [5]]. As a result, many patients continue to experience acute pathological fractures. For a proportion of these patients, the pathological fracture serves as the initial symptom of their MBD, adding to the already substantial stress and anxiety associated with a cancer diagnosis [6,7]. To meet the urgent demand for enhanced risk assessment and timely interventions in patients with MBD, it is essential to investigate innovative approaches that consider diverse factors beyond the known clinical indicators.

In recent years, there has been growing recognition of Social Determinants of Health (SDOH) and their influence on health outcomes across different medical disciplines [8].

SDOH are non-medical factors such as education level, employment status, marital status, and ethnicity [9] Patients with poor SDOH have been found to experience higher readmission rates, increased healthcare costs, and poorer general health in orthopedics [8,9]. Patients with poor SDOH face challenges when accessing healthcare services, and lower levels of trust and reduced access to the healthcare system have been associated with socioeconomic status and ethnicity [[10], [11], [12]]. Poor SDOH factors may contribute to patients not routinely seeking care, and therefore, are potentially at risk of developing MBD without knowing. When patients are unaware of their MBD, it may put them at risk of sustaining an acute pathologic fracture as no preventive measures such as bisphosphonate administration or prophylactic surgery can be considered. Moreover, lack of awareness can lead to lower survival due to the delayed detection of their primary cancer [15,16]. Despite the established importance of SDOH in orthopedics, the specific relationship between SDOH and perioperative survival of MBD remains unexplored.

Therefore, this study aimed to investigate in a group of patients with long-bone metastases requiring surgery: (1) whether SDOH factors are associated with post-operative survival and (2) whether SDOH factors are associated with clinical presentation of an acute pathological fracture as the initial symptom of MBD.

## 2 Methods

### 2.1 Study design and setting

The institutional review board of study center granted approval to waive the need for informed consent in this retrospective observational study, which took place at two urban tertiary care centers specializing in orthopaedic oncology affiliated within one healthcare entity in the northeastern United States. This study was performed in accordance with the Strengthening the Reporting of Observational studies in Epidemiology (STROBE) guidelines (Appendix 1) [17].

### 2.2 Participants

**Fig. 1:**
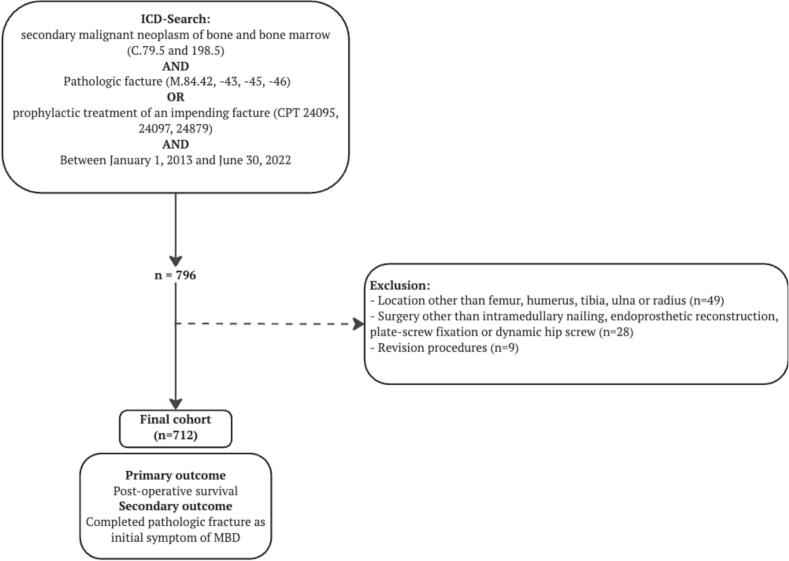
Flowchart visualizing inclusion of patients. ICD = International Classification of Disease; CPT = Current Procedural Terminology.

We included all 712 consecutive patients that were 18 years or older who received surgical treatment for a completed or impending pathological fracture due to long-bone metastases between January 1st, 2013, and June 30th, 2023, from a local prospectively kept database [18,19]. The exclusion criteria were as follows; (1) patients who received previous surgical treatment for a metastatic lesion of a long-bone, (2) surgical treatment other than intramedullary nailing, *endo*-prosthetic reconstruction, dynamic hip screw, or plate screw fixation, or (3) revision procedures. These exclusions ensured a homogenous cohort of patients undergoing primary surgical treatment, as prior surgeries such as alternative non-standard procedures, and revision cases could introduce variability in treatment effects, prognosis, and outcomes. A multidisciplinary team of a medical oncologist, anesthesiologist, and orthopedic surgeon were responsible for the assessment of patients’ ability to undergo surgery (Fig. 1). No a priori sample size calculation was conducted. Instead, all eligible patients within the available dataset were included to maximize statistical power and reflect real-world conditions.

### 2.3 Outcome measures and variables

The primary outcome of our study was overall post-operative survival, which was defined as the time from the date of surgery until death by any cause. The last date of follow-up was June 30th, 2023, allowing for at least 1-year of follow-up, with a median follow-up of 272 days (IQR, 84–794 days). Sixty-four patients were lost to follow-up in the first year and were censored in statistical analysis. The secondary outcome of our study was presentation with a completed pathologic fracture as the first symptom of MBD. This was defined as time from date of diagnosis of completed or impending pathologic fracture to date of surgery of less than 7 days. Clinical notes and radiology reports were manually reviewed to determine whether these patients had a prior diagnosis of MBD in the axial skeleton or long bones before their pathological fracture. A multidisciplinary team consisting of orthopedic surgeons, oncologists, radiotherapists, and radiologists assessed patients using the Mirels’ score alongside individual factors, such as surgical eligibility, to determine the need for surgical intervention. The Mirels score evaluates four criteria—tumor location, pain level, lesion size, and lesion type (blastic, lytic, or mixed)—with a total score ranging from 4 to 12. In general, we considered a score of 9 or higher as a high risk of fracture, warranting surgical consideration and was therefore considered an impending fracture.

Three independent reviewers (TG, AL, CJ) manually extracted the following variables from electronic health records: age, sex, body mass index (BMI), SDOH factors [8], Eastern Cooperative Oncology Group (ECOG) performance score [20], presence of visceral metastases, presence of brain metastases, pre-operative use of systemic therapy, primary tumor histology grouping as being either a slow-growth, moderate growth, or rapid growth type of tumor according to the primary tumor histology grouping of Katagiri et al. [21], radiographic characteristics, grouped in lytic lesion, blastic lesion, and mixed (both lytic and blastic) lesions, as well as ten pre-operative laboratory values (Fig. 1). SDOH factors included (1) marital status; (2) employment status; (3) smoking status; (4) education status; (5) insurance status; (6) ethnicity and (7) the Area Deprivation Index (ADI) on both national and state level [22]. Importantly, we chose to retain subgroups with missing SDOH-factors including employment status, education level, insurance coverage, and smoking status. These variables were derived from hospital intake questionnaires and are subject to nonresponse bias, meaning that non-response to these questionnaires might be an indicator for lower socio-economic status.

Primary and secondary insurance coverage were analyzed as separate variables to capture the complexity of the U.S. healthcare system, in which individuals frequently rely on multiple layers of insurance to access care. Primary insurance typically serves as the main payer for healthcare services, while secondary insurance may cover additional costs such as co-payments, deductibles, or services not fully reimbursed. This distinction is particularly relevant in the U.S. context, where insurance type and depth of coverage have been shown to influence access to diagnostics, treatment timelines, and clinical outcomes [23].

The ADI was extracted using the patients’ zip-code and includes various factors that influence socio-economic wellbeing such as income, education, employment, and housing quality. The ADI is a validated measure used to assess neighborhood-level socioeconomic disadvantage. It combines data from multiple socioeconomic indicators, such as income, education levels, employment rates, and housing quality, to create a composite score that reflects the overall deprivation of a specific area (Supplemental Table 1). Both state and national ADI-scores were collected since most – but not all – patients were residents of the state of Massachusetts. State ADI scores range from 1 to 10, where a score of 1 indicates the least social deprivation. Meanwhile, national ADI scores range from 1 to 100, with a score of 1 indicating the least social deprivation.

### 2.4 Description of study population

In total, 712 patients were included in the study, of which 324 (46 %) were male. The median age was 65 years (IQR 58–73). Most patients were treated for a metastatic lesion of the femur (525 patients [75 %]), followed by the humerus (140 patients [20 %]) and the tibia (19 patients [3 %]) The median ADI was 21 (IQR 13–29) on a national level and 5 (IQR 3–6) on a state level. Most patients were married or had a life partner (450/712, 65 %), while 113/712 (16 %) were single, divorced, or legally separated, and 62/712 (9 %) were widowed. Regarding smoking status, 261/712 (37 %) had never smoked, 260/712 (37 %) were former smokers, 67/712 (9 %) were current smokers, and 124/712 (17 %) had unknown or declined to provide their smoking status. Education level consisted of 235 (34 %) patients who graduated from college and 175 (25 %) who graduated from high school. Employment status included 299 (43 %) retired patients, 218 (31 %) who were unemployed or had unknown employment status, 97 (14 %) who worked full-time, and 98 (14 %) with other types of employment. Regarding insurance, 545 (78 %) patients had registered insurance, including 445 (64 %) with private insurance, 81 (12 %) with government insurance, 16 (2 %) with social insurance, and 3 (<1%) with other types of insurance.

Overall survival in days was 264 (IQR 79–772), with a 30-day mortality of 7 % (47/712), 90-day mortality of 24 % (168/712), and 1-year mortality of 50 % (354/712). Clinical presentation with a completed pathologic fracture as the initial symptom of MBD occurred in 15 % (106/712) of patients. Of the total cohort, 596 (85 %) patients had known MBD including 251 (43 %) patients with a completed fracture and 339 (57 %) patients with an impending fracture. (see Table 1).

**Table 1:** Baseline characteristics of the study population (n = 712).

Clinical factors	Median (IQR) | N (%)
Age	66 (58–73)
BMI	26 (23–30)
Male sex	324 (46)

**Primary tumor group**	
Slow growth	260 (37 %)
Moderate growth	222 (31 %)
Rapid growth	230 (32 %)
Pre-operative systemic therapy	423 (59 %)
Pre-operative chemotherapy	388 (54 %)
Pre-operative targeted therapy	167 (23 %)
Pre*-*operative SERM therapy	102 (14 %)
Pre*-*operative use of bisphosphonates	144 (20 %)
Pre*-*operative use of denosumab	45 (6 %)
Presence of brain metastases	114 (16 %)
Presence of visceral metastases	316 (44 %)
Pathologic fracture	365 (51 %)

**ECOG performance score**	
0	130 (18 %)
1	335 (47 %)
2	140 (20 %)
3	98 (14 %)
4	9 (1 %)

**Radiographic characteristics**	
Lytic	
Blastic	
Mixed	

**Location**	
Femur	542 (76)
Humerus	146 (21)
Tibia	20 (2.8)
Ulna	2 (<0.01)
Radius	2 (<0.01)

**Area Deprivation Index**	
State level	5 (3–6)
National level	21 (13–29)

**Marital status**	
Married/life partner	466 (65)
Single/divorced	178 (25)
Widowed	68 (10)

**Smoking status**	
Never	261 (37)
Former	260 (37)
Every day	67 (9)
Declined/unknown	124 (17)

**Education**	
Graduated − post graduate	60 (8.4)
Graduated − college	243 (34)
Associates degree	7 (1)
Some college	60 (8.4)
Graduated − high school	181 (25)
Less than high school	13 (1.8)
Other	28 (3.9)
Declined/unknown	34 (4.8)

Employment*	
Retired	307 (43)
Employed	141 (20)
Not employed	74 (10)
Disabled	55 (7.7)
Homemaker	6 (0.8)
Declined/unknown	129 (18)

**Insurance (primary)**	
Medicare	282 (40)
Private	214 (30)
Medicaid	44 (6.2)
Specialized	9 (1.3)
Unknown	157 (22)

**Insurance (secondary)**	
Medicare	18 (2.5)
Private	237 (33)
Medicaid	52 (7.3)
Specialized	25 (3.3)
Unknown	369 (52)

**Outcomes**	
In-hospital mortality	18 (2.5)
30-day mortality	47 (6.6)
90-day mortality	168 (24)
1-year mortality	354 (51)
Survival (days)	265 (79–773)
Pathologic fracture presentation	106 (15)

Values were demonstrated as N(%), or Mean (SD)/Median (IQR) based on their normality using the Shapiro-Wilk test. BMI = Body Mass Index; ADI = Area Deprivation Index.

### 2.5 Missing data

The MissForest algorithm was employed for imputation to address missing data [24]. MissForest is a non-parametric, machine-learning-based imputation method that uses random forest models to estimate missing values iteratively. The algorithm leverages observed data from other variables to predict and replace missing values, ensuring that the imputed data aligns with the relationships within the dataset. In this study, variables with missing data included use of denosumab (31 %), use of bisphosphonates (31 %), alkaline phosphatase (30 %), ECOG-performance status (29 %), albumin (28 %), absolute lymphocyte count (24 %), absolute neutrophil count (24 %), radiographic characteristics (17 %), calcium (12 %), hemoglobin (11 %), sodium (11 %), white blood cell count (11 %), and BMI (5.3 %). For SDOH factors, missing data was not imputed. Instead, cases with unknown SDOH factor values were treated as a distinct category and included as their own variable, since “unknown” values in questionnaires may carry unique implications for survival and clinical presentation [25].

### 2.6 Statistical analysis

Categorical variables were presented as count (%) and continuous data as median with interquartile ranges (IQR) depending on non-parametric distribution tested using the Shapiro-Wilk test. A Cox Proportional Hazards model, adjusted for key confounding factors (including age, ECOG performance status, and laboratory values such as pre-operative hemoglobin, pre-operative albumin), was used to evaluate the independent impact of various factors on postoperative survival. We assessed the proportional hazards assumption of the Cox Proportional Hazards model using Schoenfeld residuals. The Schoenfeld test indicated that the assumption was satisfied for all covariates (p > 0.05), suggesting that the hazard ratios remained constant over time [26]. Collinearity was assessed using variance inflation factors (VIF), and no values exceeding 10 were found. Therefore, no further adjustments for multicollinearity were necessary. Multivariate logistic regression was performed to calculate hazard-ratios and 95 % confidence intervals (CI) for variables found to be associated with clinical presentation of a pathologic fracture in univariate analysis. The regression model was adjusted for general demographics, clinical factors, treatment factors and laboratory values.

A predefined subgroup analysis was performed in patients with the most common primary tumors (breast, renal, and lung cancer; n = 353) to assess whether associations between clinical/social variables and outcomes were consistent across tumor types. Separate multivariable Cox regression and logistic regression models were constructed using the same modeling strategy as in the full cohort. Due to the smaller number of events in this subgroup, the number of covariates in the fracture presentation model was further reduced to avoid model overfitting. Model assumptions for proportionality and multicollinearity were tested and met as described above.

All continuous variables were standardized, meaning that the difference between the mean of the total cohort and the variable value of the patient was divided by the standard deviation. Doing so, the hazard ratios of all continuous variables are in proportion to each other.

P-values of < 0.05 were considered significant*.* All statistical analyses were performed using Python’s statsmodels package, and sci-kit learn and pandas libraries (Python Software Foundation, Wilmington, DE, USA).

## 3 Results

### 3.1 Post-operative survival

Multivariate Cox regression analysis revealed that certain social determinants of health (SDOH) were associated with overall survival. A trend toward worse survival was observed among single individuals compared to those married or living with a partner (HR 1.21, 95 % CI 0.97–1.51; p = 0.09), while widowed patients showed a similar non-significant association (HR 1.33, 95 % CI 0.95–1.85; p = 0.09). Patients with unknown or unemployed status demonstrated a trend toward poorer survival compared to employed individuals (HR 1.30, 95 % CI 0.97–1.74; p = 0.08). Other employment categories including retired, disabled, homemaker were not significantly associated with survival (all p > 0.05). Black patients had significantly improved survival compared to White patients (HR 0.58, 95 % CI 0.34–0.97; p = 0.04), while other ethnicities showed no statistically significant differences. No significant associations were found between overall survival and education level, smoking status, insurance coverage, or the Area Deprivation Index (all p > 0.05) (Supplemental Table 1).

### 3.2 Acute clinical presentation of a pathological fracture as the first symptom of MBD

In multivariate analysis, higher ECOG performance status was the only clinical variable significantly associated with presenting with a pathologic fracture, indicating increased odds with worsening functional status (OR 1.25, 95 % CI 1.00–1.56; p = 0.049). Patients with secondary insurance coverage were significantly less likely to present with a fracture (OR 0.26, 95 % CI 0.14–0.49; p < 0.01). Attending college was associated with a trend toward decreased odds of pathologic fracture at presentation, though this did not reach statistical significance (OR 0.64, 95 % CI 0.41–1.01; p = 0.054). Other social determinants—including area deprivation index, marital or employment status, and smoking—were not significantly associated with fracture risk (p > 0.05). Likewise, tumor growth rate, BMI, sex, laboratory values, radiographic lesion characteristics, and use of targeted therapy or SERM were not independently associated with acute fracture presentation (p > 0.05). Only receipt of pre-operative chemotherapy remained strongly protective (OR 0.34, 95 % CI 0.21–0.56; p < 0.01) (Supplemental Table 2).

### 3.3 Post-operative survival and clinical presentation in patients with common primary tumors

In a sub-analysis of patients with the most common primary tumors (breast, renal, and lung cancer; n = 353), clinical and social factors showed varying associations with fracture presentation and overall survival.

Regarding post-operative survival in the same subgroup, ECOG performance status remained a strong independent predictor of worse survival (HR 1.34, 95 % CI 1.15–1.57; p < 0.01). Among tumor types, patients with lung cancer had significantly worse survival compared to those with breast cancer (HR 1.63, 95 % CI 1.01–2.64; p = 0.047). Several laboratory parameters were independently associated with survival, including lower albumin (HR 0.70, 95 % CI 0.55–0.90; p < 0.01), lower hemoglobin (HR 0.75, 95 % CI 0.59–0.95; p = 0.01), and lower sodium (HR 0.73, 95 % CI 0.58–0.91; p < 0.01). Among social determinants, Black patients had significantly improved survival compared to White patients (HR 0.58, 95 % CI 0.34–0.97; p = 0.04), while no significant associations were observed for other racial or socioeconomic variables (Supplemental Table 3).

From the multivariate model for pathologic fracture at presentation, attending college was significantly associated with a reduced likelihood of presenting with a pathologic fracture (OR 0.54, 95 % CI 0.30–0.95; p = 0.03). No other social determinants or clinical variables reached statistical significance in the multivariate model (Supplemental Table 4).

## 4 Discussion

Pathologic fractures increase the burden on patients and healthcare systems. SDOH factors such as socioeconomic status and ethnicity influence health outcomes but their impact on MBD is unknown [8,13,14]. In this study, SDOH factors such as single marital status and unknown employment status were associated with decreased post-operative survival. Worse performance score was a significant predictor of fracture occurrence, while higher education levels and secondary insurance coverage were SDOH-factors associated with lower likelihoods of acute fracture presentation. These findings suggest potential protective effects of socioeconomic resources and education on fracture risk.

Clinically, the presence of rapid tumor growth, unfavorable ECOG performance scores high platelet counts, and elevated alkaline phosphatase levels were significantly associated with presentation with acute completed pathologic fractures.

### 4.1 Limitations

This study has several limitations. First, its retrospective design limited us in safeguarding control over the quality and accuracy of the data collected, as data was missing in some variables. Variables such as food insecurity status and household income were not available to us retrospectively and were therefore not included in the analysis. To minimize missing data, an extensive manual electronic chart review was conducted. Patients diagnosed before 2013 were excluded from the analysis because the ADI, developed in 2015, does not include data from before 2013. Second, this study may have been subject to selection bias, as patients treated conservatively for metastatic lesions were excluded. However, the number of patients presenting with pathologic fractures who are managed conservatively is relatively low in our institution. Therefore, their exclusion is unlikely to have a meaningful impact on the results. Third, this study was conducted in two urban tertiary care centers in the northeastern United States, which may not be representative of rural or community healthcare settings or healthcare institutions in other parts of the world. Differences in resources and patient demographics might limit the applicability of findings to other populations.

Nonetheless, this study is the first to find an association between adverse effects of SDOH factors on post-operative survival and clinical presentation in orthopedic oncology patients, using a large institutional cohort while adjusting for numerous confounding clinical variables.

### 4.2 Post-operative survival

The findings of the current study reveal a nuanced relationship between specific SDOH indicators and post-operative survival.

Although not significant, marital status showed a trend toward being a predictor of post-operative survival which highlights the importance of social support systems in health outcomes. Having a partner may facilitate earlier recognition of symptoms, better adherence to treatment, and improved post-operative care, which could collectively contribute to better survival outcomes [27,28]. Conversely, the absence of such support may delay medical intervention and increase vulnerability to poorer outcomes.

The Area Deprivation Index (ADI), a neighborhood-level metric for socioeconomic disadvantage, was not associated with survival. While ADI is a well-validated measure of community-level deprivation, its lack of association in this study aligns with prior research suggesting that area-level deprivation measures such as ADI may not fully capture individual-level socioeconomic risk in certain patient populations.

### 4.3 Presentation with a pathologic fracture as the first symptom of MBD

Patients who had attended tertiary education were less likely to present with acute completed pathologic fractures. This association remained statistically significant in our subgroup multi variate analysis of patients with breast, renal, and lung cancer, suggesting a consistent relationship between higher educational attainment and earlier presentation. This may be attributed to higher health literacy among educated individuals, enabling them to seek medical attention earlier when symptoms arise. They are more likely to understand risk factors, adhere to routine checkups, and seek early interventions for conditions like MBD. For instance, Raghupathi et al. demonstrated that adult education levels, especially tertiary education, was a critical indicator influencing healthcare in terms of infant mortality, life expectancy, child vaccination rates, and enrollment rates [8,29,30]. Furthermore, Guo et al. examined the effect of education on treatment adherence in a cardiologic population. Education was indirectly associated with treatment adherence, as patients who had received a higher education more often had social support and a better understanding of health information, enabling them to make informed decisions about their care and follow medical instructions accurately [31]. Patients with lower education levels may not have the necessary knowledge and understanding to connect symptoms, and take the necessary steps, to maintain their health. This may result in delayed diagnosis and treatment [32]. It could be beneficial to create targeted educational interventions designed to be understood by patients with limited health literacy, although its effects on preventing MBD progression in these patients is not certain [33].

In the full cohort, patients with secondary insurance coverage were also less likely to present with acute completed pathologic fractures. In the U.S. healthcare system, individuals often rely on a combination of primary and secondary insurance to access care. Primary insurance (e.g., Medicare, Medicaid, or employer-based private insurance) is the main payer for healthcare services, while secondary insurance can provide additional financial coverage for out-of-pocket costs such as deductibles, co-payments, and services not fully covered by the primary insurer. This dual coverage structure may improve access to diagnostics, specialty consultations, and earlier interventions that help detect and manage metastatic bone disease (MBD) proactively.

This protective association is supported by prior research showing that patients with limited insurance – especially patients who are insured through Medicaid – experience reduced access to outpatient orthopedic care, with urban practices often less willing to accept Medicaid due to lower reimbursement rates [34,35]. In addition to enabling better healthcare access, secondary insurance may also serve as a proxy for broader socioeconomic advantages, such as stable employment, higher income, or improved health literacy. These factors likely influence both the timing and frequency of healthcare utilization, reducing the likelihood of delayed presentation with advanced skeletal disease.

Patients with poorer functional status, as indicated by higher ECOG performance scores, were more likely to present with acute pathologic fractures. This likely reflects delayed recognition of symptoms in individuals with limited mobility or greater baseline impairment, where new-onset skeletal pain or reduced function may be attributed to existing disability or comorbidities rather than underlying metastatic disease [36]. For patients with decreased performance status, reduced physical activity may also mask early symptoms of bone involvement, leading to missed opportunities for early diagnosis. Moreover, impaired functional reserve may increase fracture risk even with low-impact events or minimal trauma [37]. However, this association was not observed in the subgroup analysis, possibly due to smaller sample size or reduced variability in functional status among patients with common tumors.

Receipt of preoperative chemotherapy was associated with a significantly reduced likelihood of presenting with a pathologic fracture as the initial manifestation of metastatic bone disease. This protective association may reflect closer oncologic surveillance in patients undergoing systemic treatment, allowing for earlier detection and management of skeletal metastases before structural compromise occurs. Patients receiving chemotherapy are typically engaged in more frequent clinical evaluations and imaging for primary tumor monitoring, which may facilitate incidental identification of bone lesions prior to fracture. Additionally, systemic therapy may exert direct antitumor effects that slow skeletal progression, further reducing fracture risk. These findings underscore the potential benefit of active cancer treatment not only for systemic control but also in mitigating acute orthopedic complications of advanced disease.

## 5 Conclusion

This study underscores the important, and thus far unknown role of SDOH in the outcome and presentation of metastatic bone disease. While marital status showed a trend toward worse survival among single patients, this association did not reach statistical significance after adjustment, suggesting that it may be less important than clinical factors in this patient population. Pathologic fractures, observed in 15 % of patients as the first symptom of MBD, were more frequent in individuals with poor performance status, and lack of secondary insurance coverage. Subgroup analyses further highlighted that education level may play a protective role in reducing acute fracture presentations in common tumor types. These findings emphasize the need for targeted strategies, such as health literacy programs, improved access to care, and early detection efforts, particularly for high-risk groups. Further research should explore the interplay between social and clinical factors to inform interventions that reduce disparities and improve outcomes for patients with metastatic bone disease.

## Ethics statement

This study was approved by our institutional review board.

## 8 Consultation

Harvard catalyst.

## Declaration of Generative AI and AI-assisted technologies in the writing process

During the preparation of this work the author(s) used ChatGPT 4o to improve grammar and readability. After using this tool/service, the author(s) reviewed and edited the content as needed and take(s) full responsibility for the content of the publication.

## CRediT authorship contribution statement

**Tom M. de Groot:** Writing – review & editing, Writing – original draft, Visualization, Validation, Software, Resources, Project administration, Methodology, Investigation, Formal analysis, Conceptualization. **Lotte R. van der Linden:** Writing – original draft, Methodology, Conceptualization. **Angad D.S. Bedi:** Data curation. **Andreea A. Lucaciu:** Methodology, Investigation, Formal analysis. **Caleb C. Jang:** Data curation. **Olivier Q. Groot:** Writing – review & editing, Writing – original draft, Supervision, Conceptualization. **Job N. Doornberg:** Writing – review & editing, Supervision. **Paul C. Jutte:** Writing – review & editing, Supervision. **Santiago A. Lozano-Calderon:** Writing – review & editing, Supervision. **J.H. Schwab:** Writing – review & editing, Supervision, Conceptualization.

## Funding

The authors report no funding disclosures for this study.

## Declaration of competing interest

The authors declare the following financial interests/personal relationships which may be considered as potential competing interests: T.M. de Groot reports financial support was provided by Foundation De Drie Lichten. T.M. de Groot reports financial support was provided by Foundation Dr Henry Muller’s National Fund. T.M. de Groot reports financial support was provided by Michael van Vloten fund. T.M. de Groot reports financial support was provided by Nijbakker Morra Foundation. If there are other authors, they declare that they have no known competing financial interests or personal relationships that could have appeared to influence the work reported in this paper.
