# Supplementary material for: Social determinants are associated with clinical presentation of acute pathological fracture in metastatic long-bone disease
Source: J Bone Oncol. 2025 Aug 5;54:100707. doi: 10.1016/j.jbo.2025.100707 (PMC12343859; doi:10.1016/j.jbo.2025.100707)
Supplement: Supplementary Table 1 [file mmc2.docx]

| **Supplemental Table 1.** Univariate and multivariate Cox Proportional Hazards regression for overall survival. | | | | |
| --- | --- | --- | --- | --- |
|  | ***Univariate*** | | ***Multivariate*** | |
| **Clinical variables** | HR (95% CI) | p-value | HR (95% CI) | p-value |
| Age | 1.06 (0.93 - 1.24) | 0.21 | - | - |
| Male sex | 1.14 (0.96 - 1.35) | 0.13 | 1.02 (0.83 - 1.25) | 0.87 |
| BMI | 0.74 (0.64 - 0.85) | **<0.01** | 0.88 (0.77 - 1.01) | 0.07 |
| Pathologic fracture | 1.25 (1.05 - 1.48) | **0.01** | 1.20 (1.00 - 1.45) | 0.06 |
| Brain metastases | 1.65 (1.32 - 2.07) | **<0.01** | 1.10 (0.85 - 1.41) | 0.48 |
| Visceral metastases | 1.57 (1.32 - 1.87) | **<0.01** | 1.24 (1.03 - 1.50) | **0.02** |
| ECOG performance score | 1.49 (1.36 – 1.62) | **<0.01** | 1.23 (1.11 - 1.37) | **<0.01** |
| **Primary tumor group** | | | | |
| Slow growth tumor | 0.53 (0.44 - 0.64) | **<0.01** | ref | ref |
| Moderate growth tumor | 1.11 (0.92 - 1.33) | 0.27 | 1.50 (1.16 - 1.94) | **<0.01** |
| Rapid growth tumor | 1.89 (1.57 - 2.27) | **<0.01** | 2.04 (1.56 - 2.66) | **<0.01** |
| **Pre-operative treatment** |  |  |  |  |
| Pre-operative chemotherapy | 1.50 (1.26 - 1.79) | **<0.01** | 1.48 (1.21 - 1.81) | **<0.01** |
| Pre-operative targeted therapy | 0.97 (0.80 - 1.18) | 0.76 | - | - |
| Pre-operative SERM therapy | 0.74 (0.58 - 0.95) | **0.02** | 0.83 (0.62 - 1.12) | 0.23 |
| Pre-operative use of bisphosphonates | 1.20 (0.98 - 1.48) | 0.08 | 1.00 (0.79 - 1.27) | 0.98 |
| Pre-operative use of denosumab | 1.48 (1.07 - 2.05) | **0.02** | 1.26 (0.88 - 1.80) | 0.20 |
| **Surgery location** | | | | |
| Femur | 1.17 (0.96 - 1.44) | 0.13 | ref | ref |
| Humerus | 0.80 (0.64 - 1.00) | 0.052 | 0.78 (0.61 - 1.00) | **0.046** |
| Tibia | 1.12 (0.68 - 1.85) | 0.65 | 0.87 (0.51 - 1.48) | 0.61 |
| Ulna | 0.35 (0.05 - 2.52) | 0.30 | 0.58 (0.08 - 4.26) | 0.59 |
| Radius | 1.33 (0.33 - 5.32) | 0.69 | 1.40 (0.33 - 5.89) | 0.67 |
| Multiple | 2.07 (0.92 - 4.63) | 0.08 | 2.50 (1.07 - 5.87) | **0.04** |
| **Lesion characteristics** | | | | |
| Lytic | 1.09 (0.91 - 1.29) | 0.34 | ref | ref |
| Blastic | 0.79 (0.58 - 1.09) | 0.15 | 0.67 (0.48 - 0.94) | **0.02** |
| Mixed | 1.20 (0.89 - 1.60) | 0.23 | 1.22 (0.89 - 1.69) | 0.22 |
| **Ethnicity** | | | | |
| White | 1.47 (1.13 - 1.91) | **<0.01** | ref | ref |
| Asian | 0.61 (0.35 - 1.09) | 0.09 | 0.63 (0.34 - 1.15) | 0.13 |
| Black | 0.59 (0.37 - 0.94) | **0.03** | 0.58 (0.34 - 0.97) | **0.04** |
| Declined or unknown | 0.90 (0.57 - 1.43) | 0.66 | 0.64 (0.38 - 1.10) | 0.10 |
| Hispanic | 0.86 (0.32 - 2.31) | 0.77 | 0.43 (0.15 - 1.20) | 0.11 |
| Other/Multiracial | 0.80 (0.44 - 1.45) | 0.46 | 0.72 (0.38 - 1.37) | 0.32 |
| **Pre-operative laboratory values** | | | | |
| Absolute lymphocytes | 1.06 (0.98 - 1.14) | 0.16 | 1.01 (0.88 - 1.16) | 0.87 |
| Absolute neutrophils | 1.10 (0.96 - 1.27) | 0.17 | 0.94 (0.76 - 1.17) | 0.58 |
| Albumin | 0.67 (0.6 - 0.76) | **<0.01** | 0.80 (0.69 - 0.93) | **<0.01** |
| Alkaline phosphatase | 1.20 (1.11 - 1.29) | **<0.01** | 1.22 (1.11 - 1.34) | **<0.01** |
| Calcium | 0.84 (0.62 - 1.14) | 0.27 | - | - |
| Creatinine | 0.99 (0.88 - 1.12) | 0.89 | - | - |
| Hemoglobin | 0.65 (0.57 - 0.75) | **<0.01** | 0.78 (0.66 - 0.91) | **<0.01** |
| Sodium | 0.62 (0.53 - 0.73) | **<0.01** | 0.69 (0.59 - 0.81) | **<0.01** |
| Platelet count | 0.95 (0.82 - 1.1) | 0.46 | - | - |
| White blood cell count | 1.15 (1.01 - 1.3) | **0.04** | 1.15 (0.89 - 1.48) | 0.28 |
| **SDOH-factors** | | | | |
| Area Deprivation Index | | | | |
| ADI state level | 0.97 (0.88 - 1.05) | 0.44 | - | - |
| ADI national level | 0.93 (0.85 - 1.01) | 0.10 | 0.98 (0.89 - 1.08) | 0.67 |
| Marital status | | | | |
| Married/life partner | 0.82 (0.69 - 0.98) | **0.03** | ref | ref |
| Single | 1.28 (1.06 - 1.56) | **<0.01** | 1.21 (0.97 - 1.51) | 0.09 |
| Widowed | 0.97 (0.72 - 1.31) | 0.83 | 1.33 (0.95 - 1.85) | 0.09 |
| Level of education | | | | |
| Post-Graduate | 0.86 (0.63 - 1.18) | 0.36 | 0.83 (0.58 - 1.18) | 0.29 |
| College Graduate | 1.01 (0.84 - 1.20) | 0.93 | ref | ref |
| Some College | 0.84 (0.62 - 1.13) | 0.25 | 0.86 (0.61 - 1.21) | 0.38 |
| High School Graduate | 0.94 (0.77 - 1.14) | 0.53 | 0.87 (0.69 - 1.10) | 0.26 |
| Some High School | 0.75 (0.43 - 1.30) | 0.30 | 0.83 (0.46 - 1.50) | 0.54 |
| Less than High School | 1.04 (0.53 - 2.00) | 0.92 | 1.07 (0.52 - 2.17) | 0.86 |
| Other education | 1.27 (0.78 - 2.06) | 0.33 | 1.16 (0.70 - 1.94) | 0.56 |
| Unknown education | 1.37 (1.07 - 1.75) | **<0.01** | 1.07 (0.77 - 1.47) | 0.69 |
| Smoking status | | | | |
| Never | 0.77 (0.65 - 0.92) | **<0.01** | ref | ref |
| Former | 1.28 (1.08 - 1.53) | **<0.01** | 1.20 (0.96 - 1.50) | 0.11 |
| Smoker | 1.39 (1.05 - 1.85) | **0.02** | 1.09 (0.77 - 1.53) | 0.64 |
| Unknown smoker | 0.83 (0.65 - 1.06) | 0.15 | 0.91 (0.67 - 1.22) | 0.51 |
| Employment status | | | | |
| Employed | 0.96 (0.78 - 1.19) | 0.72 | ref | ref |
| Disabled | 0.98 (0.72 - 1.33) | 0.90 | 0.86 (0.59 - 1.26) | 0.44 |
| Homemaker | 0.73 (0.27 - 1.96) | 0.54 | 0.74 (0.26 - 2.06) | 0.56 |
| Retired | 0.91 (0.76 - 1.08) | 0.27 | 0.86 (0.66 - 1.13) | 0.28 |
| Unknown or unemployment | 1.21 (0.99 - 1.46) | 0.06 | 1.30 (0.97 - 1.74) | 0.08 |
| Primary insurance present | 0.91 (0.73 - 1.12) | 0.37 | - | - |
| Secondary insurance present | 0.91 (0.77 - 1.09) | 0.31 | - | - |
| ADI = Area deprivation index; BMI = Body mass index; SERM: Selective estrogen receptor modulators; ECOG = Eastern cooperative oncology group; ref = reference; OR = Odds Ratio; CI = Confidence Interval; SDOH = Social Determinants of Health; Continuous variables were standardized, meaning that the difference between the mean of the total cohort and the variable value of the patient was divided by the standard deviation. Doing so, the hazard ratios of all continuous variables are in proportion to each other.  **Bold** p-values indicate statistical significance of p<0.05.  *All primary tumors were categorized as either slow growth, moderate growth or rapid growth tumors per the Katagiri primary tumor histology.(22) Slow growth tumors include: hormone dependent breast cancer, hormone dependent prostate cancer, malignant lymphoma, malignant myeloma, and thyroid cancer; moderate growth tumors include: hormone independent breast cancer, non-small cell lung cancer with molecularly targeted therapy, hormone independent prostate cancer, renal cancer, sarcoma, gynaecological cancer, and other tumors; rapid growth tumors include: other lung cancer, colon/rectal cancer, gastric cancer, liver cancer, pancreatic cancer, head and neck cancer, other urological cancer, esophageal cancer, melanoma, cervical cancer and gallbladder cancer. | | | | |
