# Supplementary material for: Social determinants are associated with clinical presentation of acute pathological fracture in metastatic long-bone disease
Source: J Bone Oncol. 2025 Aug 5;54:100707. doi: 10.1016/j.jbo.2025.100707 (PMC12343859; doi:10.1016/j.jbo.2025.100707)
Supplement: Supplementary Table 2 [file mmc3.docx]

| **Supplemental Table 2.** Univariate and multivariate Cox Proportional Hazards regression for clinical presentation with a pathologic fracture (n=712). | | | | |
| --- | --- | --- | --- | --- |
|  | ***Multivariate*** | | ***Multivariate*** | |
| **Clinical variables** | OR (95% CI) | p-value | OR (95% CI) | p-value |
| Age | 1.48 (1.07 - 2.03) | **0.02** | 1.14 (0.81 - 1.60) | 0.45 |
| Male sex | 1.11 (0.73 - 1.69) | 0.62 | - | - |
| BMI | 1.05 (0.79 - 1.40) | 0.73 | - | - |
| Brain metastases | 0.58 (0.30 - 1.12) | 0.11 | - | - |
| Visceral metastases | 0.99 (0.65 - 1.51) | 0.97 | - | - |
| ECOG performance score | 1.32 (1.07 - 1.62) | **0.01** | 1.25 (1.00 - 1.56) | **0.049** |
| **Tumor characteristics** |  |  | - | - |
| Slow growth tumor | 0.66 (0.42 - 1.05) | 0.08 | ref | ref |
| Moderate growth tumor | 0.72 (0.45 - 1.14) | 0.16 | 0.96 (0.54 - 1.72) | 0.89 |
| Rapid growth tumor | 1.91 (1.25 - 2.92) | **<0.01** | 1.62 (0.95 - 2.77) | 0.08 |
| **Radiographic characteristics** | | | | |
| Lytic | 1.47 (0.94 - 2.28) | 0.09 | 1.05 (0.66 - 1.68) | 0.83 |
| Blastic | 0.67 (0.28 - 1.60) | 0.37 | - | - |
| Mixed | 0.97 (0.46 - 2.03) | 0.94 | - | - |
| **Pre-operative treatment** | | | | |
| Pre-operative chemotherapy | 0.30 (0.19 - 0.47) | **<0.01** | 0.34 (0.21 - 0.56) | **<0.01** |
| Pre-operative targeted therapy | 0.46 (0.26 - 0.84) | **0.01** | 0.98 (0.50 - 1.89) | 0.95 |
| Pre-operative SERM therapy | 0.60 (0.30 - 1.19) | 0.14 | - | - |
| **Laboratory values** | | | | |
| Absolute lymphocyte count | 0.80 (0.26 - 2.40) | 0.69 | - | - |
| Absolute neutrophil count | 1.11 (0.89 - 1.37) | 0.36 | - | - |
| Albumin | 0.81 (0.61 - 1.08) | 0.15 | - | - |
| Alkaline phosphatase | 0.96 (0.72 - 1.29) | 0.81 | - | - |
| Calcium | 1.20 (0.89 - 1.62) | 0.23 | - | - |
| Creatinine | 0.77 (0.45 - 1.32) | 0.34 | - | - |
| Hemoglobin | 0.92 (0.68 - 1.24) | 0.59 | - | - |
| Sodium | 0.81 (0.59 - 1.11) | 0.18 | - | - |
| Platelet count | 1.34 (1.03 - 1.75) | **0.03** | 1.25 (0.94 - 1.67) | 0.13 |
| White blood cell count | 1.15 (0.93 - 1.42) | 0.19 | - | - |
| **SDOH-factors** | | | | |
| ADI state level | 1.02 (0.94 - 1.10) | 0.70 | - | - |
| ADI national level | 1.00 (0.98 - 1.01) | 0.68 | - | - |
| White race | 0.99 (0.55 - 1.79) | 0.98 | - | - |
| Married/life partner | 0.62 (0.41 - 0.94) | **0.03** | 0.76 (0.48 - 1.19) | 0.23 |
| Employed | 0.96 (0.57 - 1.62) | 0.87 | - | - |
| Attended college | 0.50 (0.32 - 0.77) | **<0.01** | 0.64 (0.41 - 1.01) | 0.054 |
| Smoking history | 0.95 (0.61 - 1.46) | 0.81 | - | - |
| Secondary insurance coverage present | 0.17 (0.14 - 0.21) | **<0.01** | 0.26 (0.14 - 0.49) | **<0.01** |
| ADI = Area deprivation index; BMI = Body mass index; ECOG = Eastern cooperative oncology group; ref = reference; OR = Odds Ratio; CI = Confidence Interval; SDOH = Social Determinants of Health; Continuous variables were standardized, meaning that the difference between the mean of the total cohort and the variable value of the patient was divided by the standard deviation. Doing so, the hazard ratios of all continuous variables are in proportion to each other.  **Bold** p-values indicate statistical significance of p<0.05. | | | | |
